# MicroRNA-155 from sputum as noninvasive biomarker for diagnosis of active pulmonary tuberculosis

**DOI:** 10.22038/ijbms.2020.44029.10324

**Published:** 2020-11

**Authors:** Hua Ying, Sun FengYing, Wu YanHong, Huang YouMing, Zhou FaYou, Zhang HongXiang, Tang XiaoLei

**Affiliations:** 1 Vascular Disease Research Center and Basic Medical Laboratory, the Second Affiliated Hospital of Wannan Medical College, Kangfu Road 10#, Wuhu 241000, Anhui Province, PR China; 2 School of Nursing, Wannan Medical College, Wenchang Xi Road 22#, Wuhu 241000, Anhui Province, PR China; 3 Department of Clinical Laboratory, the Second Peoples’ Hospital of Wuhu city, Jiuhua Zhong Road 259#, Wuhu 241000, Anhui Province, PR China; 4 Department of Microbiology, Wannan Medical College, Wenchang Xi Road 22#, Wuhu 241000, Anhui Province, PR China

**Keywords:** Biomarker, Diagnosis, microRNA-155, Pulmonary tuberculosis, Sputum

## Abstract

**Objective(s):**

Tuberculosis (TB), caused by *Mycobacterium tuberculosis*, is a widespread infectious disease around the world. Early diagnosis is always important in order to avoid spreading. At present, many studies have confirmed that microRNA (miRNA) could be a useful tool for diagnosis. This study aimed to evaluate whether miRNAs could be regarded as a noninvasive diagnosis biomarker from sputum for pulmonary tuberculosis (PTB).

**Materials and Methods:**

The *M. tuberculosis* strain H37Rv was incubated and cultured with human macrophage line THP-1. The total RNA was extracted from the THP-1 cells for detection. Six increased expressions of miRNAs were selected by miRNA microarray chips and the miRNAs were confirmed by qRT-PCR in the *M. tuberculosis* infection cell model. At last, the efficiency of other methods was compared with using miRNA.

**Results:**

Only miR-155 showed a better diagnostic value for PTB than the other five miRNAs to distinguish PTB from non-PTB, including pneumonia, lung cancer, and unexplained pulmonary nodules. Next, we detected and analyzed the results of 68 PTB patients and 122 non-PTB, the sensitivity and specificity of miR-155 detection was 94.1% and 87.7%, respectively. It was higher than sputum smear detection and anti-TB antibody detection. But slightly lower than ELISpot (97%, *P=*0.404). Interestingly, the ranking of sputum smear by Ziehl-Neelsen staining had positive correlation with the expression level of miR-155 in smear-positive sputum (R^2^=0.8443, *P*<0.05).

**Conclusion:**

Our research suggested that miR-155 may be an efficiency biomarker for active PTB diagnosis and bacteria-loads evaluation.

## Introduction

Tuberculosis (TB), caused by various strains of mycobacteria, mainly *Mycobacterium tuberculosis* in humans, is a very contagious infectious disease (1). As one of the most deadly infectious diseases to seriously threaten human health, TB can attack multiple organs, especially the lungs causing pulmonary TB (PTB), but can also invade other parts of the body causing extra-PTB (2). Most infections are asymptomatic and latent, but about ten percent of PTB patients infected with *M. tuberculosis *eventually develop active disease which, in case of delayed treatment, kills over fifty percent of those infected (3). Therefore, early diagnosis of PTB is the key to reducing mortality; however, diagnosis remains a challenge. 

At present, the methods of TB diagnosis mainly have direct sputum smear staining for acid-fast bacilli (AFB), sputum culture, tuberculin skin test (TST), and chest radiography in routine clinical practice (4). However, direct sputum smear staining has a low sensitivity and *M. tuberculosis* culture, the gold standard for the diagnosis of *M. tuberculosis*, has the drawbacks of being time-consuming and low success rate of culture (4-6). In infants of less than six years, the TST is used while this test is not specific for *M. tuberculosis *detection. The chest radiography has high technical requirements for operators and high cost and, it is not suitable for children and pregnant women. In view of the above, these conventional methods of TB diagnosis have certain disadvantages that cause diagnostic delays and misdiagnosis, increasing the rate of morbidity and mortality. Therefore, discovering new methods to effectively improve the diagnosis of PTB is urgently needed. 

MicroRNAs (miRNAs) are small single-stranded noncoding RNAs (generally 19~25 nucleotides in length) that play a vital role in many important biological processes, such as cell proliferation, cell migration, cell invasion, cell apoptosis, cell death, and early embryonic development, through regulating the expression of their target mRNAs (7-9). Except for being highly conserved in variety of species, miRNAs have remarkable stability in different kinds of biological specimen, including serum, plasma, sputum, urine, fresh snap-frozen materials, formalin-fixed, and even in very harsh conditions, this is due to their resistance to extreme pH and temperatures, repeated freeze-thaw cycles, long storage in frozen conditions, and endogenous or exogenous RNA enzymes (10). Owing to these prominent characteristics, miRNAs can be considered as good candidates for use as biomarkers in human diseases. Recently, miRNAs as potential diagnostic biomarkers of multiple diseases have been widely studied (11-14).

Ndzi *et al*. reported that plasma miR-29a-3p showed good diagnostic performance (84.35%) between active TB and latent TB and good distinguishing performance (81.37%) between active TB and control groups (15). Serum and sputum miR-144 levels were up-regulated significantly in TB patients, the ROC analysis of serum miR-144 showed high sensitivity (99.19%) and specificity (94.87%), the ROC analysis of sputum miR-144 also showed high sensitivity (86.29%) and specificity (99.15%), indicating the potential application value of serum and sputum miR-144 levels in the diagnosis of TB (16). Zheng *et al*. reported that plasma miR-155 was significantly up-regulated in TB patients and showed a high area under curve (AUC) value of 0.976, obtaining a good diagnostic performance in discriminating PTB from healthy controls (17). Taken together, the above results indicated that fluid miRNAs show a good diagnostic performance in TB patients.

Compared with other samples, sputum is one of the most conveniently and noninvasively accessible biological fluids. Growing evidence has suggested that sputum miRNAs could be a promising diagnostic biomarker for early detection of many human diseases, such as chronic obstructive pulmonary disease and lung cancer (18-20). However, at present, there is little research on sputum miRNA in the diagnosis of tuberculosis. Therefore, considering the above advantages of sputum miRNAs, the study aimed to detect *M. tuberculosis* induced differentially expressed miRNAs from host macrophage and explore the potential diagnostic value of sputum miR-155 in the diagnosis of active PTB. 

## Materials and Methods

Sixty-eight patients with active PTB (39 males and 29 females) were enrolled from the Second Peoples’ Hospital of Wuhu City and the Second Affiliated Hospital of Wannan Medical College, China, from January 2017 to October 2018. The subjects ranged in age from 19 to 78 years (mean age 46.8±28.3 years). According to the revised international definitions in TB control of the World Health Organization (WHO) (21), the inclusion criteria of patients with active PTB were as follows: 1. typical PTB clinical symptoms such as fever and cough, 2. fibrocavitary lung infiltrate on chest computed tomography (CT) and chest X-ray, 3. at least one positive sputum examination results (sputum smear or sputum culture), 4. detection results of ELISpot was positive. Patients were excluded who had other coexisting lung diseases. During the same time period, 50 healthy volunteers (25 males and 25 females) who attended the physical examination, were recruited as healthy controls. The subjects ranged in age from 27 to 74 years (mean age 52.4±14.7 years). Healthy donors had no history of TB infection, no organic diseases, and no clinical symptoms of any infectious disease. Meanwhile, three pulmonary disease groups with total of 72 patients were included: pneumonia, lung cancer, unexplained pulmonary nodules. In conclusion, the three basic characteristic of 190 participants, including 122 non-PTB donors and 68 PTB patients, were described in this study (Table 1).

The study was approved by the ethics committee of the Second Peoples’ Hospital of Wuhu City and the Second Affiliated Hospital of Wannan Medical College and carried out in compliance with the Helsinki Declaration. Written informed consent was obtained from all participants and their families before the commencement of the study.

***Sputum samples collection***

Early morning sputum samples, 1.5~2.5 ml sputum without saliva, were collected into a sterile plastic container (nuclease-free), and processed within 1 hr before anti-TB treatment and at 1 month after treatment. Obtained sputum was processed with 0.1% dithiothreitol and 20 U/ml RNase inhibitor for homogenization. The homogenized sputum samples were centrifuged at 4000 rpm at 4 ^°^C for 20 to 30 min and the supernatant was collected, subpackaged into 1.5 ml Eppendorf tubes without RNase, and then stored in liquid nitrogen immediately for later testing. To exclude unqualified sputum specimens, at least two sputum smears were prepared for Wright-Giemsa staining. The cells in the sputum smears were counted and classified under a low-magnification microscope. The specimen qualified for the study if the percentage of squamous cells was less than 80% (22, 23).

***THP-1 cells culture***

Human macrophage cell line THP-1 (GDC100) was kindly provided by professor Xiao-Lian Zhang (Wuhan University, Wuhan, China). THP-1 cells were routinely grown in RPMI 1640 (Life Technologies) supplemented with 10% Fetal Bovine Serum (FBS, Wisent Inc., Quebec, Canada) and induced differentiation by the addition of 100 nM phorbol 12-myristate 13-acetate (PMA) (Beyotime Biotechnology, Shanghai, China) to the culture medium for 48 hr at 37 ^°^C. 

***M. tuberculosis culture and infection model***

*M. tuberculosis* H37Rv (strain American Type Culture Collection (ATCC) 93009) was purchased from the Beijing Biological Product Institute (Beijing, China) (24). 120 clinical *M. tuberculosis* strains were obtained from Hainan Agricultural Reclamation Hospital, Dongfang People’s Hospital, and Sanya People’s Hospital. *M. tuberculosis* strains were cultivated in Middlebrook 7H9 (Roche) containing 10% albumin dextrose catalase (ADC, Roche) for three weeks at 37 ^°^C, and harvested while in the log phase of growth and resuspended with RPMI 1640 culture medium. 

For the *M. tuberculosis* infection model, THP-1 cells were seeded and cultivated in 6-well plates to reach approximately 80% confluent monolayer cells. Then, the cells were washed in PBS and the fresh culture medium was added. THP-1 cells were infected with *M. tuberculosis* strains at a multiplicity of infection (MOI) of 100 (cells: bacteria=1:100) for 12 hr, 24 hr, and 48 hr, respectively as experimental groups and then washed by PBS three times to remove extracellular *M. tuberculosis*. Cells without *M. tuberculosis* strains infection were cultivated for indicated time periods as control groups. Then, cells were collected in TRIzol reagent (Invitrogen, California, USA) and preserved at -80 ^°^C until RNA isolation. 

***RNA isolation***

Total RNA was extracted from THP-1 cells with *M. tuberculosis* infection and sputum supernatant using TRIzol reagent (Invitrogen) and further purified with an RNeasy mini kit (Qiagen, USA) according to the manufacturer’s protocol. The quantity of the extracted RNA was detected by a NanoDrop2000 spectrophotometer (NanoDrop Technologies, USA). The purity and integrity were assessed using denaturing agarose gel electrophoresis. 

***Microarray analysis***

miRNA expression profile in RNA samples from THP-1 cells with *M. tuberculosis* infection and THP-1 cells without *M. tuberculosis* infection was detected by Human miRNA Expression Microarray Release 14.0 (Agilent Technologies, Inc.). The experiment and quality control were performed by CapitalBio Corp (Beijing, China) according to the manual. Fluorescence images on the chips were obtained using luxScan10K Microarray Scanner (CapitalBio) and digitized using Array-Pro image analysis software (Media Cybernetics) for data analysis. Different expressions of miRNAs were selected based on a two-fold change of increase or decrease between TB patients group and non-TB participants group.

***Quantitative real-time PCR (qRT-PCR)***

Total RNA samples (1 μg each group) were reverse transcribed into cDNA using the miScript^®^ II RT Kit (Qiagen, Valencia, CA, USA), according to the manufacturer’s protocols. Specific primers for qRT-PCR amplification of different genes were purchased from Qiagen (Hilden, Germany). The primer sequences are listed in Table 2. qRT-PCR analysis was performed on ABI Step One Real-Time PCR System (Applied Biosystems; Thermo Fisher Scientific, Inc.) in a total volume of 20 μl, containing 2 μl of synthesized cDNA solution, 500 nM of each primer, 10 μl of 2× miScript SYBR-Green PCR Mix (Qiagen), and added sterile water to 20 μl. The amplification conditions included 95 ^°^C for 5 min, followed by 42 cycles of 95 ^°^C for 15 sec, and extension at 58 ^°^C for 30 sec. Each assay was performed in duplicate, and the expression fold change of each miRNA was calculated according to the 2^-ΔΔCt ^method (25) and normalized to the housekeeping U6 expression. Differences in expression levels between two groups (PTB and non-PTB group, patients with active PTB group and healthy group) were measured using the Student’s t-test using SPSS 20.0 with *P*<0.05 considering statistically significant. 

***ELISpot assay***

Peripheral blood mononuclear cells (PBMCs) were separated by centrifugation from 5 ml heparinized blood samples and then added into the pre-coated ELISPOT plate. The experiment was performed using ELISpot kit (Dakewe Biotech Co., Ltd, Shenzhen, China) according to the manufacturer’s instructions. The number of spot-forming cells (SFCs) was counted by an automated ELISpot reader (Cellular Technology Ltd., Shaker Heights, OH) in each well. 

***Acid-fast staining test (Ziehl-Neelsen staining)***

At least two sputum smears were prepared on clean glass slides and were subjected to acid-fast staining using an acid-fast staining kit (Y-S Biotechnology, Shanghai, China) according to the manufacturer’s instructions.

***TB antibody test***

The serum was separated by centrifugation (3500 rpm for 5 min ) from a 3 ml blood sample. The experiment was performed using a TB antibody test kit (Nattbio Co., Ltd, Guangzhou, China) according to the manufacturer’s instructions. 

***Statistical analysis***

Data were recorded as the mean±standard deviation (±SD) and analyzed by IBM SPSS Statistics version 20.0 software (IBM Corp., Armonk, NY, USA). ANOVA test or Student’s t-test was used for statistical analysis. The chi-square test was performed for comparing results between the sputum miRNA-155 detection and other test methods. *P*<0.05 was regarded as significantly different. 

## Results

***Different expression of miRNAs in THP-1 cells with H37Rv treatment***

The miRNA microarray chips were used to analyze the expression of 36 miRNAs in THP-1 cells with *M. tuberculosis* H37Rv strain infection. The results showed that only two miRNAs were down-regulated, while most of miRNAs were up-regulated in THP-1 cells infected with H37Rv. Among them, the expression levels of six miRNAs had a significant up-regulation: miR-155, miR-29b-1*, miR-150, miR-146a, miR-212, and miR-483-5p in THP-1 cells with *M. tuberculosis* infection for 12 hr, 24 hr, and 48 hr. The up-regulation of these six miRNAs was time-dependent. Compared with the uninfected group, the up-regulation of miR-155 and miR-29b-1* was statistically significant (F=17.95 and 58.18, all *P*-values were <0.01) (Table 3).

***Differential expression of miRNAs in THP-1 cells infected with clinical isolated M. tuberculosis strains***

**Figure 1:**
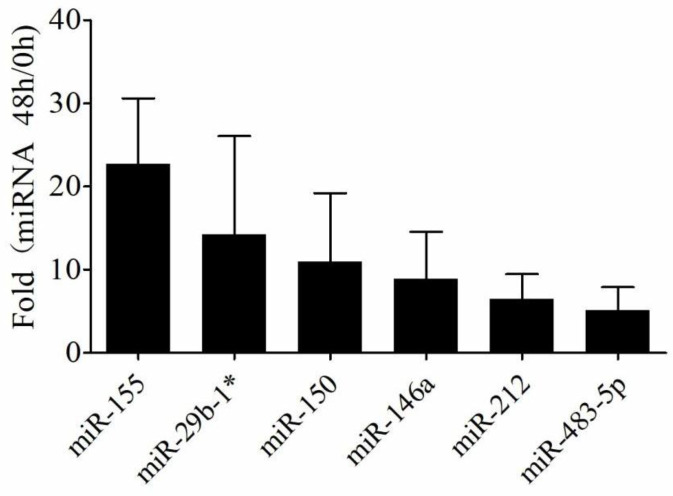
Differential expression of miRNAs in THP-1 cells infected with clinical isolated *Mycobacterium tuberculosis* strains

Expression levels of the above six miRNAs were analyzed by qRT-PCR in THP-1 cells infected with *M. tuberculosis* strains, isolated from 120 clinical sputum samples. The data showed that the expression levels of the six miRNAs (miR-155, miR-29b-1*, miR-150, miR-146a, miR-212, and miR-483-5p) were up-regulated, which was consistent with the above findings. In addition, the up-regulated multiple of miR-155 was higher than the other 5 miRNAs (all *P-*values were <0.01) (Figure 1). Thus, miR-155 was selected as the diagnostic target for further study.

***The relative level of six kinds of miRNA in sputum from 190 donors***

**Figure 2:**
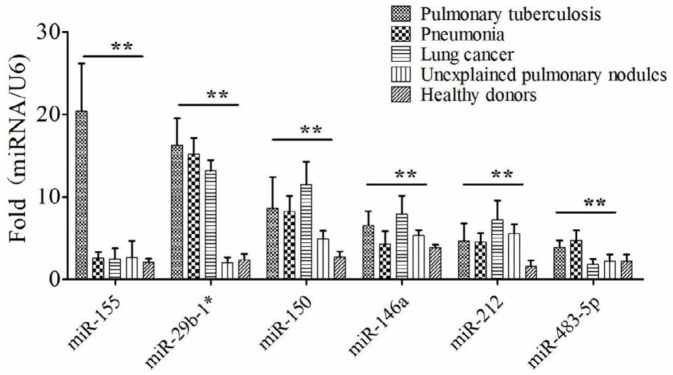
The relative level of miRNA-155 from six kinds of miRNAs be worthy for Pulmonary tuberculosis (PTB) diagnosis in clinical sputum

The total RNA was extracted from 190 sputa and the relative level of miRNAs was detected via qRT-PCR. The 190 donors were from five groups, 68 patients with PTB, 32 patients with pneumonia, 28 patients with lung cancer, 12 patients with unexplained pulmonary nodules, and 50 healthy donors (Table 1). The six kinds of miRNAs showed different detection performances among the five groups (Figure 2). miR-155 showed a better detection performance to distinguish PTB patients from non-PTB donors. Although there were statistical differences between PTB and non-PTB using the other five kinds of miRNAs, they had lower value than miRNA-155 for PTB diagnosis from sputum. So miRNA-155 was selected and used to be a detection tool in the next experiment. 

***Diagnostic value of sputum miR-155 for active PTB***

**Figure 3:**
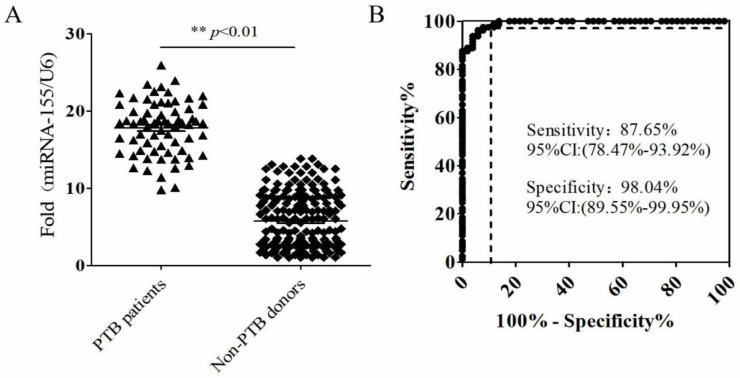
MiR-155 showed a better diagnostic value for active PTB from sputum

**Figure 4:**
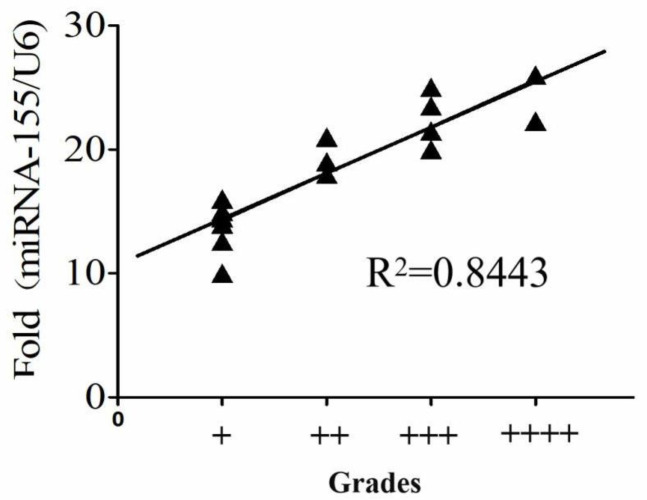
The good correlation between the grade of Ziehl-Neelsen staining and the level of miR-155 in sputum smear-positive

The qRT-PCR was used to analyze the expression levels of miR-155 in sputum specimens from 68 active PTB patients and 122 non-PTB patients (32 patients with pneumonia, 28 patients with lung cancer, 12 patients with unexplained pulmonary nodules, and 50 healthy donors) (Table 1 and Figure 3A). Then, we used the receiver operating characteristic curve (ROC curve) to evaluate the sensitivity and specificity of sputum miR-155 for active PTB diagnosis. Results showed that the sensitivity of sputum miR-155 detection was 94.1% (95% CI:86.18%-96.45%), the specificity was 87.7% (95% CI:76.13%-94.38%) (Figure 3B). The positive rate of sputum miR-155 detection was 94.1%, which was higher than sputum smear detection (22.1%, *P*<0.001) and anti-tuberculosis antibody detection (83.8%, *P*<0.05). But it was slightly lower than ELISpot (97%, *P*=0.404) (Table 4). Interestingly the ranking of sputum smear was positively correlated with the expression level of miR-155 in sputum (R^2^=0.8443, *P*<0.05) (Figure 4).

**Table 1:** The basic information of 190 donors in this research for clinical verification

**N** **ame**	**P** **rimer sequence**
hsa-miR-155	Forward 5’-ACGCTCAGTTAATGCTAATCGTGATA-3’
	Reverse 5’-ATTCCATGTTGTCCACTGTCTCTG-3’
hsa-miR-29b-1*	Forward 5’-GGGTAGCACCATTTGAAATC-3’
	Reverse 5’-TTTGGCACTAGCACATT-3’
hsa-miR-150	Forward 5’-TAGACGCGTCCGCGAGAGA-3’
	Reverse 5’-GTGCAGGGTCCGAGGT-3
hsa-miR-146a	Forward 5’-ACACTCCAGCTGGGTGAGAACTGAATTCCATG-3’
	Reverse 5’-TGTCGTGGAGTCGGCAATTC-3’
hsa-miR-212	Forward 5’-GCGCTGGAATGTAAAGAAGT-3’
	Reverse 5’-GTGCAGGGTCCGAGGT-3’
hsa-miR-483-5p	Forward 5’-ACACTCCAGCTGGGAAGACGGGAGGAAAGAA-3’
	Reverse 5’-CTCAACTGGTGTCGTGGA-3’

F: female; M: male

**Table 2:** The six pairs of primers sequence for qRT-PCR detection in this research

**miRNAs**	**12** **hr (** **X±SD)**	**24** **hr (** **X±SD)**	**48** **hr (** **X±SD)**	***F*** **, ** ***P-*** **value**
**miR-155**	6.25±1.23	13.56±2.31	22.71±5.22	17.95, 0.0029
miR-29b-1*	5.11±1.54	17.26±1.09	19.94±2.47	58.18, <0.000
**miR-150**	4.62±0.94	4.28±1.41	6.01±1.62	1.376, 0.3223
**miR-146a**	3.26±0.88	3.81±1.02	4.29±0.83	0.954, 0.436
**miR-212**	3.14±0.65，	3.94±0.55	4.01±0.74	1.672, 0.264
**miR-** **483-5p**	2.76±0.39	2.98±0.24	3.46±0.38	3.257, 0.110

**Table 3:** Differential expression of six miRNAs in THP-1 cells with H37Rv infection at 3 time points

**Groups (n)**	**P** **ulmonary** **tuberculosis** **(68)**	**P** **neumonia ** **(32)**	**Lung cancer** **(28)**	**Unexplained pulmonary nodules** **(12)**	**Healthy donors** **（50）**
**Age(** **X±S)**	46.8±28.3	56.9±29.4	61.5±20.6	41.8±17.6	52.4±14.7
**F/M (n/n)**	39/29	19/13	18/10	3/9	25/25
**Smoking (n)**	47	14	21	1	32
**BMI**	19.9±5.6	22.9±7.8	16.7±6.1	23.9±6.8	24.1±7.7
**HIV co-infection(n)**	1	0	0	0	0

Expression levels of the above six miRNAs were analyzed by qRT-PCR in THP-1 cells infected with *Mycobacterium tuberculosis* strains, isolated from 120 clinical sputum samples, respectively. The data showed that the expression levels of the six miRNAs (miR-155, miR-29b-1*, miR-150, miR-146a, miR-212 and miR-483-5p) were up-regulated, which was consistent with the above finding. In addition, the up-regulated multiple of miR-155 was higher than other 5 miRNAs (all *P-*values were <0.01). Thus, miR-155 was selected as the diagnostic target for further study

The relative level of six kinds of miRNAs from were detected from five groups’ sputum via qRT-PCR respectively, 68 patients with pulmonary tuberculosis (PTB), 32 patients with pneumonia, 28 patients with lung cancer, 12 patients with unexplained pulmonary nodules and 50 healthy donors. The miR-155 showed a better detection performance to distinguish PTB patients from non-PTB donors. Although there were statistical differences between PTB and non-PTB using other five kinds of miRNAs, them were less value than miRNA-155 for PTB diagnosis from sputum

The expression levels of miR-155 in sputum specimens from 68 active Pulmonary tuberculosis (PTB) patients and 122 non-PTB patients (32 patients with pneumonia, 28 patients with lung cancer, 12 patients with unexplained pulmonary nodules and 50 healthy donors) respectively. The receiver operating characteristic curve (ROC curve) we used to evaluate the sensitivity and specificity of sputum miR-155 for active PTB diagnosis. Results showed that the sensitivity of sputum miR-155 detection was 94.1% (95% CI:86.18%-96.45%), the specificity was 87.7% (95% CI:76.13%-94.38%)

The detection data of 15 patients with Pulmonary tuberculosis (PTB) were analyzed the correlation between Ziehl-Neelsen staining and miR-155. From this results, the expression level of miR-155 risen as the grade increasing. The four grades of smear detection had a good correlation with miRNA-155 (R^2^=0.8443)

**Table 4:** Comparison of diagnostic values of different methods for active Pulmonary tuberculosis (PTB)

**M** **ethods**	**N** **umber of positive cases detected (n)**	**D** **etection rate (%)**	^2^**, *P-*value**
**s** **putum miR-155 detection**	64	94.1	—
**s** **putum smears** **staining**	15	22.1	72.515，.000**
**TB antibody test**	57	83.8	3.672, 0.045*
**ELISpot **	66	97	0.697, 0.404

## Discussion

In some areas of China, TB epidemic has increased in recent years, due to the dual infection of TB and HIV, the spread of drug-resistant TB, and the expansion of the floating population, which has resulted in increased health care costs and other socioeconomic burdens (26, 27). Macrophages constitute the first line of immune defense against *M. tuberculosis* infection. Previous studies indicated that miRNA expression profiles of macrophages showed differences between *M. tuberculosis* infection groups and controls (28, 29). Consistent with the previous study, we found some differential expression miRNAs in macrophages with *M. tuberculosis* H37Rv strain infection. Among them, the expression levels of six miRNAs (miR-155, miR-29b-1*, miR-150, miR-146a, miR-212, and miR-483-5p) had a significant up-regulation, and the up-regulation of miR-155 and miR-29b-1* was statistically significant (F=17.95 and 58.18, all *P*-values were <0.01). Next, we used qRT-PCR to verify the differential expression of six miRNAs in THP-1 cells with clinical isolated *M. tuberculosis* strains. The data was consistent with the above finding.

In clinical practice, TB diagnosis usually depends on clinical symptoms, sputum culture, TST, and radiography. However, these routine clinical diagnostic methods have many disadvantages and make them difficult to meet clinical needs. Therefore, an easy, rapid, and accurate diagnostic method for tuberculosis diagnosis is urgently needed. Mounting studies have indicated that miRNA expression profiles in biological fluids can reflect altered physiological and/or pathological conditions (30, 31). Moreover, miRNAs are stably present in sputum (32, 33) and the differential expression of sputum miRNAs is closely associated with multiple human diseases. For instance, expression levels of miR-629-3p, miR-142-3p, and miR-223-3p are up-regulated in the sputum of patients with severe asthma and are related to neutrophilic airway inflammation, indicating that these sputum miRNAs contribute to this asthma inflammatory phenotype (34). miR-223 was increased in sputum samples of non-small cell lung cancer (NSCLC) patients compared to healthy controls. Sputum miR-223 detection showed 82% sensitivity and 95% specificity with AUC at 0.90 in the detection of NSCLC, suggesting that sputum miR-223 could be a useful biomarker for NSCLC diagnosis (35). Zheng *et al*. reported for the first time that miRNA expression profiles in sputum were dramatically different between PTB and healthy controls, which supports the potential application for improving the diagnosis of TB (36). 

In this work, our data suggested that the expression levels of miR-155 were significantly up-regulated in THP-1 cells infected with *M. tuberculosis* H37Rv strain and clinical isolated *M. tuberculosis* strains, respectively. Previous studies also have shown that miR-155 was up-regulated in CD14^+^ or PBMCs cells from patients with active TB (37, 38). miR-155 as a crucial regulator of innate or adaptive immune responses plays an important role in host immune response against *M. tuberculosis* infection (39, 40). Considering the vital role of miR-155 in TB and prominent features of sputum miRNAs in the assessment of human diseases, we hypothesized that sputum miR-155 expression is altered in active PTB patients and could be the potential application for active PTB diagnosis. Consistent with the expected results, the expression of miR-155 was significantly increased in the sputum of patients with active PTB. ROC analysis showed that the sensitivity of sputum miR-155 detection was 94.1% and the specificity was 87.7%. Sputum miR-155 quantification obtained a good diagnostic performance in discriminating active PTB from healthy controls, which was higher than sputum smear detection (22.1%, *P*<0.001) and anti-TB antibody detection (83.8%, *P*<0.05). But it was slightly lower than ELISpot and the difference was not statistically significant (97%, *P*=0.404). In addition, interestingly the ranking of sputum smear was positively correlated with the expression level of miR-155 in sputum (R^2^=0.8443, *P*<0.05). There were some inadequacies in this study because of the limited number of donors, the 190 participants were distributed in the adult population and Han nationality. There was no verification for TB diagnosis at different ages and nationalities, especially ethnic minorities. Of course, we will expand the scope of research in the next experiment.

In conclusion, sputum miR-155 detection maybe a novel non-invasive biomarker for rapid and accurate diagnosis of 68 adult patients with active PTB.

## Conclusion

Our research suggests that miR-155 is a potential biomarker for tuberculosis diagnosis in sputum. This miRNA detection method showed higher sensitivity and specificity of diagnostic potency than other traditional methods. Especially, the sputum sample was obtained easily and conveniently for most people, due to non-invasive sampling. Of course, this target will be detected using a rapid and visualized method in our next experiment. 
